# Safety Evaluation and Flow Modification in the Anterior Cerebral Artery after Pipeline Embolization Device Deployment across the Internal Carotid Artery Terminus

**DOI:** 10.1155/2021/6657595

**Published:** 2021-08-21

**Authors:** Chao Xu, Pei Wu, Jianyi Han, Bowen Sun, Chunlei Wang, Shancai Xu, Bin Luo, Xinjian Yang, Qingchun Mu, Huaizhang Shi

**Affiliations:** ^1^Department of Neurosurgery, The First Affiliated Hospital of Harbin Medical University, Harbin, Heilongjiang Province, China; ^2^Department of Neurosurgery, Binzhou People's Hospital, Binzhou, Shandong Province, China; ^3^Beijing Neurosurgical Institute, Beijing Tiantan Hospital, Capital Medical University, Beijing, China; ^4^Department of Interventional Neuroradiology, Beijing Neurosurgical Institute and Beijing Tiantan Hospital, Capital Medical University, Beijing, China; ^5^Department of Neurosurgery, The People's Hospital of Gaozhou, Maoming, Guangdong Province, China

## Abstract

**Method:**

The clinical and imaging data of PEDs in the postmarket multicenter registry study (PLUS) in China were retrospectively analyzed, and patients were divided into two groups on the basis of the follow-up angiographic results: group 1 (no significant change in A1 blood flow) and group 2 (A1 occlusion or decreased blood flow). We collected patients' baseline data and evaluated the following imaging indicators: diameter and ratio of bilateral A1, M1, and internal carotid artery (ICA) vessels before stenting and the ratio of the PED size (sPED) to the ipsilateral ICA (I-ICA) diameter on the implantation side.

**Results:**

A total of 1171 patients were included, of whom 48 met the inclusion criteria (17 in group 1 and 31 in group 2). In group 2, three patients experienced neurological deterioration at follow-up. From the univariate analysis of outcomes, single PED without coils, incomplete aneurysm occlusion (IAO), maximum aneurysm diameter, aneurysms involving the ICA bifurcation (ICAb), and large sPED/I-ICA diameter ratio were included in the multivariate analysis (*P* < 0.20). The multivariate regression analysis results showed that the ratio of sPED/I-ICA diameter was the factor influencing A1 vessel occlusion. The area under the ROC curve was 73.2%. When the sPED/I-ICA diameter ratio was 1.14, sensitivity was 70.6%, and specificity was 77.4%.

**Conclusion:**

When an oversized PED is placed from M1 to the ICA, the higher porosity formed at the covered A1 orifice is conducive to maintaining stable A1 blood flow and reducing the risk of A1 vessel occlusion. This trial is registered with ClinicalTrials.gov identifier: NCT03831672.

## 1. Introduction

Pipeline embolization devices (PEDs) are an alternative treatment for complex intracranial aneurysms; however, there remains a risk of occlusion of jailed side branches, even in patients with good efficacy with antiplatelet drug therapy [1, 2]. Previous PED manufacturers' instructions suggested that these devices are suitable for large or giant wide-necked aneurysms from the petrous segment of the internal carotid artery (ICA) to the proximal superior hypophyseal artery in patients aged ≥22 years. Recently, the FDA incorporated ICA bifurcation into PED treatment coverage [3]. However, for carotid bifurcation aneurysms, it is often necessary to locate the distal end of the PED into the M1 segment and to cover the origin of the ipsilateral A1 segment to ensure PED stability [3–6]. Previous studies have reported that this approach may affect A1 blood flow and that A1 segment occlusion can lead to neurological deficits [3–6].

To date, few studies have evaluated the long-term prognostic impact of covering the ipsilateral A1 segment origin and the related factors leading to occlusion. We retrospectively analyzed the clinical and imaging data from the postmarket multicenter registry study (PLUS) to investigate the long-term prognostic impact of A1 occlusion and factors associated with occlusion.

## 2. Method

### 2.1. Design and Process

**Figure 1:**
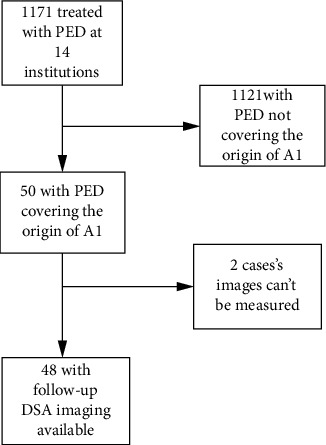
Patient selection for our study.

We performed a retrospective review using the databases of the 14 participating Chinese institutions for aneurysms treated with PEDs between 2015 and 2019 in the PLUS. The study was a consecutive, real-world cohort registry study. Local institutional review boards or ethics committees approved the study and the use of the patients' data. All operations were performed with written informed consent. Inclusion criteria were as follows: (1) for intracranial aneurysms treated with a PED, a single PED was deployed from the M1 segment to the ICA and covered the orifice of A1 and (2) follow-up digital subtraction angiography (DSA) imaging data. Exclusion criteria were as follows: (1) multiple PEDs covering the beginning of A1, (2) loss of the A1 artery on the PED treated side, and (3) no follow-up DSA imaging data. Data on the patients' general information, aneurysms involving the ICA bifurcation, maximum aneurysmal diameter, intraoperative and postoperative complications, and the mRS scores before and at the last follow-up were collected. Patient selection for our study is summarized in Figure 1.

### 2.2. Angiographic Follow-Up

Aneurysm occlusion was classified using the O'Kelly-Marotta (OKM) grading scale [7, 8]. We described the flow modification in the ipsilateral A1 segment as follows: (1) no change in flow regarding patency and (2) occlusion or diminished flow [4]. To compare changes in A1 vessels, we divided patients into two groups. Group 1 comprised patients who showed no flow changes in the jailed artery at the last angiographic follow-up, and group 2 comprised patients in whom occlusion or diminished flow in the jailed artery had been observed at the last angiographic follow-up.

### 2.3. Perioperative Management

All patients received an antiplatelet regimen that included aspirin (100 or 300 mg daily) and clopidogrel (75 mg daily). Patients who were identified as clopidogrel nonresponders received aspirin (100 mg daily) and ticagrelor (90 mg twice daily). All patients demonstrated optimal platelet activity suppression before PED placement. Board-certified neuroendovascular surgeons performed all procedures. Intravenous heparin was administered intraprocedurally to achieve an activated clotting time of >250 seconds. Heparin was discontinued after completing the procedure. Dual antiplatelet therapy was continued for 6 months after PED placement. Patients received aspirin 100 mg/d for life.

### 2.4. Parameter Measurement

All vessel measurements were obtained on the original calibrated DSA images using the Radiant DICOM Viewer 5.0.2 (https://www.radiantviewer.com/) by two experienced neurosurgeons. Measurements of the ipsilateral A1 segment and the M1 segment, supraclinoid ICA, and the contralateral A1 segment 3 mm from the ICAb were evaluated before PED deployment. For aneurysms at the ICA bifurcation (ICAb) site, a vessel diameter of 3 mm around the aneurysm was measured. The widest diameter on the calibrated images was recorded for each vessel of interest. The PED size/ipsilateral-ICA (sPED/I-ICA) ratio was defined as the ratio of the standard diameter of the PED to the average diameter of the proximal M1 segment and the landing area in the ICA. Other ratios, such as ipsilateral-A1/ipsilateral-M1 (I-A1/I-M1), ipsilateral-A1/ipsilateral internal carotid artery (A1/I-ICA), ipsilateral-M1/ipsilateral internal carotid artery (I-M1/ICA), and the odds ratio of ipsilateral-A1/contralateral-A1 (I-A1/C-A1), were obtained after the sPED/I-ICA measurement.

### 2.5. Statistical Analysis

Categorical variables are reported as proportions, and continuous variables are reported as the mean ± standard deviation or the median and interquartile range, as appropriate. Between the groups, the variables were compared using the chi-square test, Mann-Whitney *U* test, and *t*-test. Univariate analysis was used to test the following independent factors: age, sex, hypertension, smoking, aneurysm size, aneurysm occlusion status, aneurysms involving the ICAb, and the following ratios: I-A1/I-M1, I-A1/I-ICA, I-M1/ICA, and the odds ratios of I-A1/C-A1 and sPED/I-ICA. Factors that were predictive in the univariate analysis (*P* < 0.20) were entered into a multivariate logistic regression analysis. *P* values < 0.05 were considered statistically significant. We created a ROC curve to test the ability of the sPED/I-ICA ratio to predict jailed A1 vessel patency. Statistical analysis was performed using SPSS 25 (IBM Corp., Armonk, NY, USA).

## 3. Results

### 3.1. General Data

A total of 48 patients were identified retrospectively in the present study; 31 (65%) patients were women, and 17 (35%) were men. The mean age was 54 ± 13.8 (range: 14–77) years. The average maximum diameter of the treated aneurysms was 9.2 ± 6.6 mm. Of the 48 patients, 12 (25%) had a history of hypertension, 14 (29.2%) had a history of tobacco smoking, and 4 (8.3%) had a history of diabetes (Table 1). Of the 51 aneurysms, 9 (30%) were fusiform. Aneurysms occurred at the following locations: 4 in the cavernous sinus segment, 13 in the ophthalmic artery segment, 16 in the posterior communicating segment, 2 in the anterior choroidal artery origin, and 12 in the ICAb; the remaining 4 aneurysms were middle cerebral artery (MCA) aneurysms. Of the 48 patients, 47 had 1 PED implanted, and 1 required 2 PEDs. The A1 origin ostium was covered by a single PED in all patients.

### 3.2. Clinical and Radiographic Outcomes

**Figure 2:**
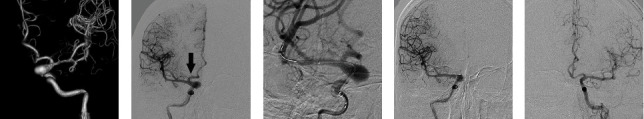
A 50-year-old male patient was treated with a single 4.25 mm × 25 mm pipeline embolization devices (PED) following the unexpected discovery of a C7 segment fusiform aneurysm by computed tomography angiography (CTA). The PED size/ipsilateral internal carotid artery (sPED/I-ICA) ratio was 1.05. The 2-year follow-up angiography (d) showed that the aneurysm still had a residual body, but A1 (b, black arrow) was occluded. The mRS score of the patient was 2. (a, b) Preoperative 3D reconstruction and orthographic angiography images showing patency of the ipsilateral A1 segment. (c) PED implantation. (d) Two-year angiography image showing the residual aneurysm with ipsilateral A1 occlusion. (e) Contralateral 2-year follow-up angiography showing that the right A2 blood supply area was compensated by the anterior communicating artery.

**Figure 3:**
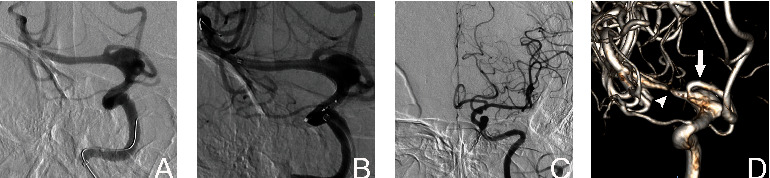
Female patient, 56 years old. CTA images showing that a C7 segment aneurysm was treated with a single PED measuring 3 mm × 25 mm; the sPED/I-ICA ratio was 1.21. The 6-month follow-up angiography showed that a residual aneurysm was present with in-stent stenosis (indicated by the white arrowhead in d), and the patient's mRS score was 2. A1 (indicated by the white arrow in d) is intact. (a, b) The A1 origin was covered before and after PED implantation. (c) Contralateral ICA angiography. (d) Six-month follow-up angiography 3D reconstruction showing that most of the residual aneurysm and A1 remained intact, but M1 shows in-stent stenosis.

mRS scores in three patients changed, and one patient's right ICA became stenotic after PED treatment; the mRS score was 1 before operation and 3 at the last follow-up. The second patient with A1 occlusion had an mRS score of 1 before operation and 2 at the last follow-up (Figure 2). The third patient had in-stent restenosis and cerebral ischemia; the mRS score was 0 before operation and 2 at the last follow-up (Figure 3). Follow-up DSAs performed a median of 201 days (interquartile range: 168 to 276 days). The O'Kelly-Marotta (OKM) grading scale grades for the treated aneurysms were as follows: 31 were OKM-D (no filling), as complete obliteration; incomplete aneurysm occlusion (IAO) occurred in 20 aneurysms: 2 were OKM-C2 (entry remnant), 11 were OKM-B2/B3 (subtotal filling), and 7 were OKM-A1/A2 (total filling). Regarding the flow modification outcomes for A1, the last follow-up angiography showed that the covered A1 segment was occluded in 11 patients (22.9%), with diminished flow in 20 patients (41.7%). There was no flow change in 17 patients (35.4%) (Table 1).

### 3.3. Changes in the A1 Segment

Group 1 (*n* = 17) comprised patients who showed no flow changes in the jailed artery. Group 2 (*n* = 31) comprised patients who had occlusion or diminished flow in the jailed artery. The mean age in group 1 was 57.7 ± 14.5 years versus 53.0 ± 13.4 years in group 2. The mean aneurysm size in group 1 was 7.1 ± 3.8 mm versus 10.9 ± 7.5 mm in group 2. Complete obliteration was achieved in 6 (35.3%) patients in group 1 versus 25 (73.5%) in group 2. The sPED/I-ICA ratio in group 1 was 1.19 versus 1.06 in group 2. Other variables are summarized in Table 1 and Table S1.

**Figure 4:**
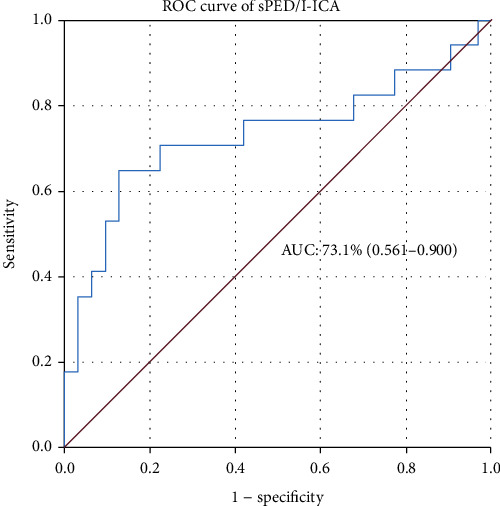
Ability of the sPED/I-ICA ratio to predict jailed A1 vessel patency. The area under the curve (AUC) for the sPED/I-ICA ratio was 73.1% (95% CI: 0.561–0.900). When the sPED/I-ICA ratio was 1.14, the sensitivity was 70.6%, and the specificity was 77.4%.

In the univariate analysis, statistically significant factors associated with group 1 versus group 2 were single PED treatment (OR: 2.566, 95% CI: 0.693–9.502; *P* = 0.158), IAO (OR: 0.196, 95% CI: 0.056–0.687; *P* = 0.011), aneurysm size (OR: 1.099, 95% CI: 0.977–1.235; *P* = 0.114), aneurysms at the ICAb (OR: 3.048, 95% CI: 0.855–10.865; *P* = 0.086), and sPED/I-ICA (OR: 0.003, 95% CI: 0.0001–0.286; *P* = 0.012). Multivariate analysis demonstrated that sPED/I-ICA (OR: 0.0001, 95% CI: 0.0001–0.164; *P* = 0.013) was a statistically significant factor associated with jailed arteries with no flow change. Detailed information is shown in Table 2. The area under the ROC curve was 73.2%. When the sPED/I-ICA diameter ratio was 1.14, the sensitivity was 70.6% and the specificity was 77.4% (Figure 4).

## 4. Discussion

ICA bifurcation is an uncommon location for a PED. To avoid PED retraction after treatment, it is necessary to deploy the end of the PED in the M1 segment and cover the origin of A1 [6, 9–11]. Sufficient preoperative evaluation is required for this off-label use, and previous reports have shown that approximately 2/3 of arteries covered by a flow diverter will become stenotic or occluded during follow-up [2]. In a recent report of 10 patients with ICAb aneurysms, all PEDs covered the origin of A1, of whom 3 patients had asymptomatic occlusion of A1 [6]. We found that the blood flow reduction and occlusion rates in A1 were 41.7% and 22.9%, respectively. To the best of our knowledge, ours is the largest study to assess changes in jailed A1s in patients with PEDs deployed across the ICAb and covering the ostium of A1.

### 4.1. Prognosis of A1 Occlusion

Most researchers believe that PED placement has a significant effect on the blood flow of covered side branches. Currently, there is a consensus that if collateral circulation is inadequate to provide sufficient compensation, patency in the jailed branches is maintained by direct flow. Otherwise, if direct or pial collateral compensation is well preserved, the jailed branch will become progressively occluded or narrowed, usually without clinical consequences [2, 12]. One patient with A1 occlusion in our study had an mRS score of 1 before operation and 2 at the last follow-up, with symptoms associated with A1 occlusion. This indicates that there is a certain risk of covering A1 with a PED, and no A1 occlusion-related symptoms have been reported before, which may be related to the small number of cases. Therefore, it is necessary to fully evaluate the anterior communicating artery patency before the operation.

### 4.2. Influence of the Diameter Ratio of the Branch Vessels

Currently, it is believed that when a PED is placed in Y-shaped vessels, the blood flow changes in covered branches are related to the diameter ratio of the two branches: the thinner the branches, the greater the blood flow resistance [13]. The effect of side branch diameter on hemodynamics was quantitatively studied by Tang et al. [14]. After PED implantation, in the side branches with a diameter of 1 mm, the blood flow velocity and pressure decreased by 35% and 3.3%, respectively. For diameters of 2.0 mm, the velocity decrease was only 7.2%, but the pressure decreased by 4.3%. The reductions of the mean volume flow rates in the side branch vessel after PED deployment were 0.4% for *d* = 1.0 mm and 6.3% for *d* = 2.0 mm [14]. Narata and de Moura [15] compared the ratios of the diameters of the two subbranches in 25 patients with bifurcation aneurysms without compensation from communicating branches, namely, 11 patients with no vessel caliber change and 14 patients with vascular occlusion or subacute occlusion. The mean value difference between the two groups was statistically significant when the ratio was <0.7 (*P* < 0.001). Further computational fluid dynamics analysis of the standard idealized model showed that a ratio of <0.65 had a statistically significantly greater effect on the wall shear area of the two branches before and after operations.

The first study evaluating occlusion following a PED covering the ipsilateral A1 segment was presented by Nossek et al. [4]; the authors found that six of seven patients who demonstrated occlusion of the A1 segment or reversal of flow from contralateral vessels manifested an average A1/M1 segment ratio of 0.58 (range: 0.29–0.76). Pujari et al. [3] reported 27 patients whose PEDs were positioned between the M1 and the supraclinoid segment of the ICA and covered the origin of A1. They found an average ratio of the patent covered A1 segments to the ipsilateral M1 of 0.71, compared with 0.50 for the occluded group. The difference using the *t*-test was statistically significant (*P* = 0.0006). In our study, the A1/M1 ratio was 0.68 in group 2 and 0.67 in group 1. Although the ratio was between 0.65 and 0.70, there was no significant difference using the *t*-test (*P* = 0.87). We believe that the current overall evaluation of A1/M1 ratios is based on a small sample of studies. With the increasing use of PEDs, we look forward to the results of larger studies.

### 4.3. Effect of PED Size

The porosity of a certain model of PED changes parabolically with increased artery diameter. Relative to the diameter of the landing artery, oversized PEDs increase in porosity and play an important role in the patency flow [16, 17]. As indicated in the in vitro study by Shapiro et al. [17], metal coverage falls rapidly with increasing device oversizing, with minimum coverage already observed when the artery is only 1 mm smaller than the nominal device diameter. Thus, even relatively modest degrees of oversizing translate into substantially lower metallic coverage. In actual treatment, the PED model chosen by neurointerventionists is slightly larger than the landing vessel diameter, but the ideal proportion is currently inconclusive, and it is worth exploring what proportion may preserve the flow in the covered vessel.

Our results showed that the sPED/I-ICA ratio in group 1 was 1.19 versus 1.06 in group 2. In the univariate analysis, sPED/I-ICA ratio (OR: 0.003, 95% CI: 0.0001–0.286; *P* = 0.012) was included in the multivariate analysis. The results of the multivariate analysis demonstrated that sPED/I-ICA (OR: 0.0001, 95% CI: 0.0001–0.164; *P* = 0.013) was a statistically significant factor predicting a jailed artery with no flow change. We believe that to match the diameter of the ICA of the landing zone, a relatively large diameter PED must be placed in the M1 segment, which has different diameters from the M1 segment to the ICA. Therefore, oversizing the PED will inevitably decrease the metal coverage where the diameter changes, with less impact on the flow into A1 [3, 4, 6].

PED porosity increases from the inner to the outer curves at each point along the vessel cross-section along a 180-degree curvature in vitro [17]. However, owing to the effects of vessel curvature and changes in diameter, the porosity at the ostium of A1 observed in vivo will be substantially more complex [18]. The sPED I-ICA ratio was statistically significantly different between our two groups, which may be related to the choice of PED model because this is based on the diameter of the ICA.

Our results are in accordance with the experimental results in swine reported by Berg et al. [19]. In their research, the authors implanted 5 × 20 mm PEDs into the carotid artery, which had diameters of 4.56 mm in case 1 and 5.32 mm in case 2, and covered the ascending pharyngeal artery, which had diameters of 2.18 mm and 2.42 mm, in cases 1 and 2, respectively. Three-month postprocedure angiography showed that patency in the ascending pharyngeal artery (APhA) in case 1 was well preserved, while the ascending pharyngeal artery in case 2 was nearly occluded. Scanning electron microscopy further confirmed the structural basis of this phenomenon and evaluated the APhA ostium 3 months after procedure regarding the difference in acute stent strut angles. In case 1, the angle corresponded to 78° with a circulating surface of 359,208 *μ*m^2^. In case 2, the angle corresponded to 59.1°, with a circulating surface of 142,937 *μ*m^2^. This indicates that the oversized PED was stretched and that the metal coverage at the branch ostium was low. However, undersized PEDs are compressed, which results in high metal coverage and not only reduces the flow into the branch vessels but also provides more scaffolds for endothelial hyperplasia, which ultimately reduces the ostium area.

The mean flow rate reductions through the jailed branches are calculated using computational fluid dynamics. In the oversized case 1, mean flow rate decreases of 14.1% occurred. In comparison, the undersized case 2 showed a reduction of 25.5% [19]. In case 2, the low wall shear stress (WSS) along the strut region that covers the ostium enabled the proliferation of neointimal cells, which led to narrowing of the jailed arterial branch. Conversely, case 1 showed increased WSS at the ostium and shear load, especially across the distal area of the jailed side branch [19]. Similar results were confirmed in the study by Iosif et al., which compared the occlusive rate of jailed vessels by the PED between an anastomotic-type arterial configuration and a terminal-type. With higher occlusive rates of the anastomotic-type jailed vessels, the mean ostial shear stress on the struts was 6.7 Pa vs. 11.3 Pa for the terminal type [12]. These results are in accordance with a previous study investigating the effect of WSS on endothelial proliferation for an open-cell stent [20]. It appears that low WSS promotes endothelial proliferation not only on the stented parent artery but also on the free segments of the stent.

Clinically, in a regression analysis of 217 covered branches in 137 patients treated with a PED by Miller and Kole [21], a relatively undersized PED was associated with long-term covered vessel stenosis and occlusion. The authors suggested that relatively undersized PEDs result in higher metal coverage at the ostium of the covered vessel, which led to decreased blood flow and long-term vessel occlusion. Our results were similar. Kole et al. [22] performed regression analysis and showed that relatively undersized PEDs in 158 aneurysms treated with a single PED were associated with aneurysm resolution. Therefore, will the PED chosen to preserve A1 blood flow have an impact on the healing of aneurysms? Our results were significantly correlated with no change in A1 in the univariate analysis, but not in the multivariate analysis. This study provides a valuable reference for future treatment for considering the effect of PED size on blood flow when the PED needs to cover A1.

### 4.4. Limitations

(1) This was a retrospective study and the number of patients was relatively small. (2) Our data were derived from a multicenter study, and there were differences in the effect of the degree of PED pushing and pulling by the operators on the mesh during PED release. (3) Because some imaging data may be incomplete in a retrospective study, it is not certain that each patient's preoperative anterior communicating compensation status was evaluated preoperatively.

## 5. Conclusion

When relatively oversized PEDs are placed between M1 and the ICA, the higher porosity formed at the ostium of A1 is beneficial for maintaining stable A1 blood flow and reducing the risk of A1 artery occlusion.

## Figures and Tables

**Table 1:** Baseline demographic data for all, group 1, and group 2 patients.

Characteristic	All	Group 1 (*n* = 17)	Group 2 (*n* = 31)	*P* value
Sex				0.52
M (%)	17 (35%)	5 (29.4%)	12 (38.7%)	
F (%)	31 (65%)	12 (71.0%)	19 (61.3%)	
Mean age in yrs	54 ± 13.8	57.7 ± 14.5	53.0 ± 13.4	0.26
Hypertension (%)	12 (25%)	6 (35.3%)	6 (19.4%)	0.30
Diabetes (%)	4 (8.3%)	2 (11.8%)	2 (6.5%)	0.52
Smoking (%)	14 (29.2%)	3 (17.6%)	11 (35.5%)	0.32
Aneurysm size (mm)	9.2 ± 6.6	7.1 ± 3.8	10.9 ± 7.5	0.08
Aneurysms at the ICAb (%)	15 (31.3%)	8 (47.0%)	7 (22.5%)	0.08
Treatment				0.15
PED (%)	32 (62.7%)	13 (76.5%)	19 (55.9%)	
PED+coil (%)	19 (37.3%)	4 (23.5%)	15 (44.1%)	
Aneurysm occlusion				0.008
Complete obliteration (%)	31 (60.8%)	6 (35.3%)	25 (73.5%)	
IAO (%)	20 (39.2%)	11 (64.7%)	9 (26.5%)	
Fate of A1				
Occlusion (%)	11 (22.9%)	—	11 (35.5%)	
Diminished flow (%)	20 (41.7%)	—	20 (64.5%)	
No flow change (%)	17 (35.4%)	17 (100%)	—	
I-A1/I-ICA (mean (SD))	0.50 (0.12)	0.49 (0.11)	0.51 (0.12)	0.74
I-M1/I-ICA (mean (SD))	0.76 (0.12)	0.77 (0.12)	0.76 (0.12)	0.78
I-A1/I-M1 (mean (SD))	0.67 (0.19)	0.67 (0.22)	0.68 (0.17)	0.88
OR I-A1/C-A1 (mean (SD))	0.92 (0.24)	0.91 (0.19)	0.93 (0.27)	0.81
sPED/I-ICA (mean (SD))	1.11 (0.16)	1.19 (0.18)	1.06 (0.12)	0.006

yrs: years; M: male; F: female; ICAb: the internal carotid artery bifurcation; PED: pipeline embolization device; IAO: incomplete aneurysm occlusion; I-A1: ipsilateral-A1; I-ICA: ipsilateral internal carotid artery; SD: standard deviation; I-M1: ipsilateral-M1; OR: the odds ratio; C-A1: contralateral-A1; sPED: the PED size.

**Table 2:** Univariate and multivariate analyses of group 1 and group 2.

Characteristic	Univariate analysis^∗^			Multivariate analysis		
	OR	95% CI	*P* value	OR	95% CI	*P* value
Single PED	2.566	0.693-9.502	0.158	0.717	0.098-5.263	0.744
IAO	0.196	0.056-0.687	0.011	0.228	0.028-1.840	0.165
Aneurysm size	1.099	0.977-1.235	0.114	1.143	0.990-1.320	0.068
Aneurysms at the ICAb	3.048	0.855-10.865	0.086	5.699	0.433-74.932	0.186
sPED/I-ICA	0.003	0.0001-0.286	0.012	0.0001	0.0001-0.164	0.013

PED: pipeline embolization device; IAO: incomplete aneurysm occlusion; ICAb: the internal carotid artery bifurcation; sPED: the PED size; I-ICA: ipsilateral internal carotid artery. ^∗^Also entered in the univariate analysis but not significant: I-A1/I-ICA, I-M1/I-ICA, and I-A1/I-M1.

## Data Availability

All the data of this study can be obtained in the article and supplementary materials.

## Supplementary Materials

Supplementary Materials Table S1: the size of the PED and the sPED/I-ICA ratio of the 48 patients.
