# Supplementary material for: Safety Evaluation and Flow Modification in the Anterior Cerebral Artery after Pipeline Embolization Device Deployment across the Internal Carotid Artery Terminus
Source: Biomed Res Int. 2021 Aug 21;2021:6657595. doi: 10.1155/2021/6657595 (PMC8405287; doi:10.1155/2021/6657595)
Supplement: Supplementary Materials — Table S1: the size of the PED and the sPED/I-ICA ratio of the 48 patients. [file 6657595.f1.docx]

| **Table S1. The size of the PED and the** **sPED/I-ICA ratio of the 48 patients** | | | |
| --- | --- | --- | --- |
| No. of Patients | Group 1 or 2 | sPED | sPED/I-ICA |
| 1 | 1 | 4.0×18 | 1.423487544 |
| 2 | 1 | 3.0×25 | 0.980392157 |
| 3 | 1 | 4.5×35 | 1.081730769 |
| 4 | 1 | 5.0×35 | 1.291989664 |
| 5 | 1 | 4.0×25 | 1.433691756 |
| 6 | 1 | 3.0×25 | 1.214574899 |
| 7 | 1 | 4.5×30 | 1.020408163 |
| 8 | 1 | 4.5×25 | 0.910931174 |
| 9 | 1 | 4.5×35 | 1.145038168 |
| 10 | 1 | 4.75×20 | 1.349431818 |
| 11 | 1 | 4.5×25 | 1.296829971 |
| 12 | 1 | 3.0×20 | 0.837988827 |
| 13 | 1 | 4.75×30 | 1.21483376 |
| 14 | 1 | 2.75×20 | 1.195652174 |
| 15 | 1 | 4.25×25 | 1.501766784 |
| 16 | 1 | 3.5×30 | 1.245551601 |
| 17 | 1 | 4.0×25 | 1.204819277 |
| 18 | 2 | 3.75×25 | 0.95177665 |
| 19 | 2 | 4.25×25 | 1.054590571 |
| 20 | 2 | 3.5×35 | 1.076923077 |
| 21 | 2 | 4.25×30 | 0.764388489 |
| 22 | 2 | 4.25×25 | 0.972540046 |
| 23 | 2 | 4.25×30 | 1.210826211 |
| 24 | 2 | 5.0×35 | 0.99009901 |
| 25 | 2 | 4.0×30 | 1.183431953 |
| 26 | 2 | 4.0×35 | 1.156069364 |
| 27 | 2 | 3.25×20 | 1.048387097 |
| 28 | 2 | 4.75×35 | 1.27688172 |
| 29 | 2 | 4.0×30 | 1.242236025 |
| 30 | 2 | 3.75×30 | 1.059322034 |
| 31 | 2 | 3.5×35 | 1.029411765 |
| 32 | 2 | 3.0×35 | 1.111111111 |
| 33 | 2 | 4.0×25 | 1.005025126 |
| 34 | 2 | 3.75×20 | 0.878220141 |
| 35 | 2 | 4.25×30 | 0.981524249 |
| 36 | 2 | 4.0×30 | 1.063829787 |
| 37 | 2 | 4.25×20 | 1.136363636 |
| 38 | 2 | 4.0×20 | 0.845665962 |
| 39 | 2 | 3.5×30 | 0.92348285 |
| 40 | 2 | 4.25×30 | 1.103896104 |
| 41 | 2 | 4.5×30 | 1.133501259 |
| 42 | 2 | 5.0×20 | 1.030927835 |
| 43 | 2 | 4.0×25 | 0.954653938 |
| 44 | 2 | 3.0×30 | 1.102941176 |
| 45 | 2 | 4.25×30 | 1.398026316 |
| 46 | 2 | 3.5×20 | 1.076923077 |
| 47 | 2 | 3.75×30 | 1.168224299 |
| 48 | 2 | 3.5×25 | 1.09375 |
| PED: pipeline embolization device, sPED: the PED size, I-ICA：ipsilateral internal carotid artery. | | | |
